# Takayasu’s Arteritis Associated with Tuberculosis in a Young Yemeni Woman

**DOI:** 10.4103/1995-705X.76804

**Published:** 2010

**Authors:** Khaled Al-Aghbari, Ahmed Al-Motarreb, Faiza Askar

**Affiliations:** Department of Medicale, Faculty of Medicine, Sana’a University, Yemen

**Keywords:** Takayasu’s arthritis, tuberculosis, tuberculous lymph node

## Abstract

Takayasu’s arteritis (TA) is an autoimmune disease that affects the big arteries. A possible relationship between TA and tuberculosis (TB) has been suggested. Both diseases have similar chronic inflammatory lesions and occasionally granulomas on the arterial walls. We report a case of TA associated with TB.

## INTRODUCTION

Takayasu’s arteritis (TA) is a disease of unknown etiology, characterized histologically by an inflammatory cell infiltrate that affects all the layers of the arterial wall, especially the aorta and its major branches. Its incidence varies between 1.2 and 2.3 cases per million per year, and it is more common in Asians than in other racial groups.[1] An exact epidemiological figure from our region is not available.

The etiology of TA is not clear. A number of features suggest an autoimmune base while others raise the question that the aortitis may be an expression of tuberculin sensitization.[2–4]

A causal relationship between TA and tuberculosis (TB) had been suggested. Both diseases show similar pathological changes in the form of chronic inflammatory lesions and, occasionally, granulomas on the arterial walls.[2] The genetic relationship between these two diseases has not been reported; however, both diseases have been associated with human leukocyte antigen (HLA) alleles, cold agglutinins and cryoglobulins during the acute phase of the illness.[5,6]

## CASE PRESENTATION

An 18-year-old female patient presented with fever and weakness in the upper limbs with right hand affection more than the left. Two years before presentation, she used to have fever in the evening and, gradually, she developed a persistent fever in the last 6 weeks without cough or weight loss.

She gave a past history of hypertension, which had been diagnosed when she was eight years old. She was regular antihypertensive medication (amlodepine and aldomet). The dosage of her medications had been decreased gradually by her physician over the last few months and were finally stopped completely based on a belief that her hypertension was spontaneously cured! She also gave a history of abdominal operation at the age of 8 years for a right suprarenal mass after discovering her hypertension, and the histolpathological report revealed fatty tissue changes and not pheochromocytoma, as was believed to be, before the operation. The hypertension was not cured after the operation and the antihypertensive medications were resumed.

There was no family history of the same illness or TB in her family members.

### General examination

The patient looks ill, pale, underweight (body weight was 45 kg, height 162 cm and BMI = 17). The right upper limb pulsation was absent while it was normal in the left arm, with high force and volume in both lower limbs. Carotid pulsations were present in both sides. Blood pressure (BP) measurement was taken in the four limbs and was recorded as the following: no BP in the right upper limb, 110/70 mmHg in the left upper limb and 200/130 mmHg in both lower limbs.

The neck examination showed a significantly enlarged right cervical lymph node measuring 3.5 cm × 2.8 cm, which was firm, not tender and not fixed to the underlying structures.

Cardiac examination showed normal heart sounds at the apex of the heart with a loud S2 at the base and systolic bruit at the left supraclavicular fossa. Other systemic examinations were within normal limits. Chest and abdomen examinations revealed no abnormal findings.

### Laboratory investigations

Hemoglobin was 9 gm/dl, ESR = 120 mm in the first hour, C-reactive protein was 20 mg/dl, total white blood cells was normal with predominant lymphocytosis and normal platelet count. The serological study for ANA, cANCA and pANCA were all negative, her liver function test, renal function test, thyroid function test and urine analysis were all within normal limits. The Mantoux test for TB was performed and it was strongly positive (25 mm).

ECG showed sinus tachycardia with left axis deviation and signs of left ventricle enlargement.

The chest x-ray and abdominal ultrasound were normal. Echocardiography showed left ventricular hypertrophy with normal other internal dimensions and normal valves. Duplex vascular ultrasound revealed total total occlusion of the right subclavian artery with poststenotic flow pattern while the left subclavian artery was significantly narrowed.

**Figure 1:**
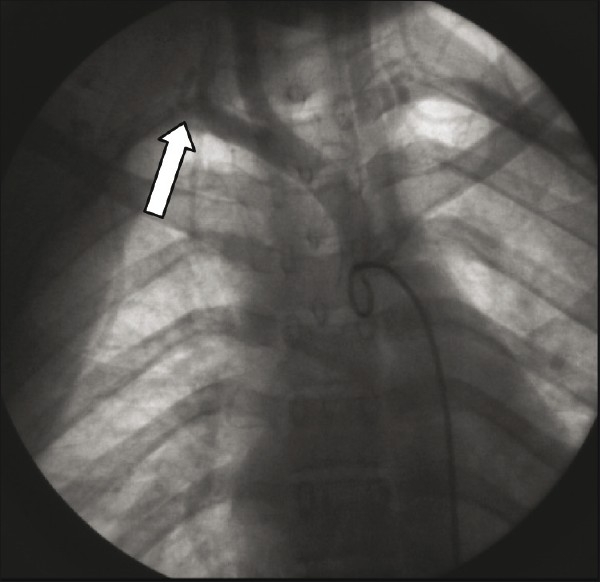
Total occlusion of the right subclavian artery

**Figure 2:**
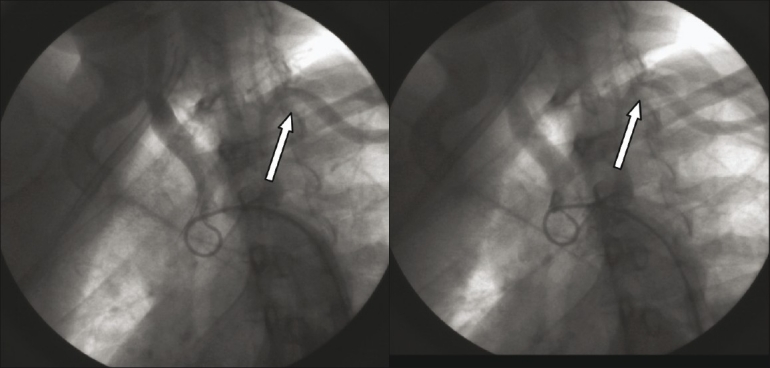
Significant stenosis of the left subclavian arteries

**Figure 3:**
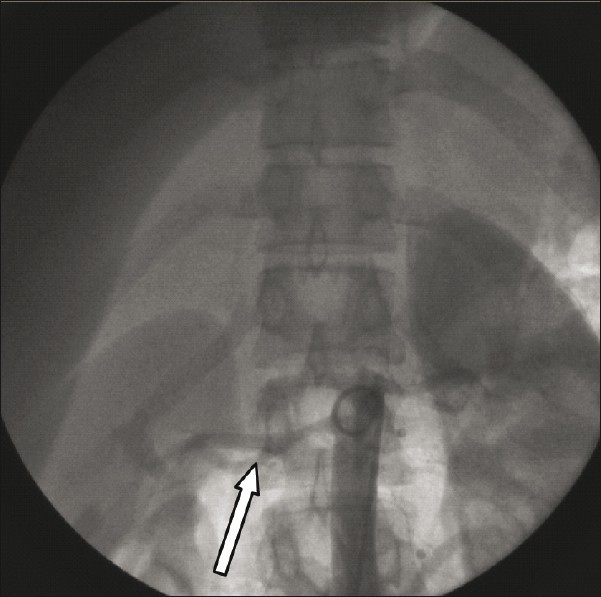
shows the total occlusion of the right renal artery

An aortogram revealed normal aortic arch with total occlusion of the right subclavian artery [Figure 1], with significant narrowing of the left subclavian artery [Figure 2]. Abdominal aorta angiography showed right renal artery stenosis with normal left renal artery [Figure 3]. It also showed good collaterals for right upper limb and a nonsignificant lesion at the origin of the left common carotid.

Histolpathological report of the right cervical lymph node biopsy showed caseating tubercles suggestive of granulomatous tuberculous lymph adenitis.

### Treatment

Standard anti-TB treatments with steroid were given in addition to antihypertensive medications and aspirin. The patient was improved significantly and the symptoms and cervical lymph nodes disappeared. She was discharged, to be followed-up in the outpatient clinic.

## DISCUSSION

TA is generally classified along with temporal arteritis as one of the giant cell arteritis.[7,8] Takayasu’s disease is one of the first vasculitides to be associated with a specific infectious agent. Despite the association with TB and the similarity between granulomatous lesions in both diseases, the exact role of *Mycobacterium tuberculosis* in the pathogenesis of TA is still unknown. Previous studies suggest that cross-reaction between Mycobacterium and human heat shock protein (HSP) might have a key role.[9,10] This hypothesis is further supported by the increased expression of 65 KDaHSPs of the involved vessels as well as the activation of subpopulations of T cells that may cross-react with the host HSP.[9,10] It has also been speculated that *M. tuberculosis* can be the triggering factor through its production of super antigens, the suggested role of which is thought to be via the stimulation of autoreactive T cells that induce vascular damage.

As a cause of renovascular hypertension, TA ranks first in white children.[11] the occurrence in a set of identical twins suggests a genetic predisposition.[12]

The presenting clinical picture in our patient was similar to a reported series in which 67–90% of the cases occurred in females with onset generally in the second and third decades.[2,3,13–15]

This patient presented with symptoms that had been found in the majority of the reported patients, including dyspnea on exertion, fever, anorexia and tachycardia.[15–18–20]

Initially, she demonstrated artheralgia and hypertension at the age of 8 years, which are commonly encountered features accompanying renovascular involvement.[11] While increased sedimentation rate was documented in 83% and 100% of two series,[13] our patient showed marked ESR elevation, which had been estimated to be 120 mm in the 1^st^ hour. The tuberculin test was strongly positive and caseating granulomatous lesions were found in the cervical lymph nodes. These findings were suggestive of an association between TB and the disease process. This connection had been reported before[21] but, in Yemen, this is the first case to be reported with documented TA and TB.

Almohammed[21] reported two cases in whom a tuberculous process was documented prior to/concomitant with TA, and both of them responded well to prednisolon and anti-TB.[21] In our patient, the vasculitis presented as an arterial hypertension years before the presentation of TB.

She was given anti-TB, steroid and antihypertensive medications. Initially, she showed a significant resolution of the cervical lymph nodes and all other systemic symptoms, but the pulse was persistently not felt in her right arm in the period of her follow-up, which extended to 1 year. The resolution of the lymph nodes and systemic manifestations might be a result of removing the triggering infectious agent or reducing the triggering antigen load, but the pulse was still absent almost because the arteritis was manifested years before the TB lymphadenitis and the changes in the vessel wall was usually permanent.

TA is a systemic vasculopathy that can progress to cause vital organ ischemia. Therefore, we gave the patient long-term and regular follow-up. ESR, C-reactive protein and duplex vascular ultrasound were carried out in each visit. TB should be kept in mind during exploration of the etiopathology of TA mainly in a region where TB is common, and we found that the use of anti-TB drugs is rationale during the treatment of this disease.

## CONCLUSION

TA is a systemic disease that might progress to cause vital organ ischemia.

Although the exact cause is not known, it is possible that an infection triggers TA in people with a genetic predisposition.
